# BasNet: Attention U-Net-Based Automated Axon Segmentation in Bielschowsky Silver-Stained Histology

**DOI:** 10.1007/s12021-026-09815-z

**Published:** 2026-09-08

**Authors:** Lukas Schönenberger, Laurin Egli, Dimitrios Gkotsoulias, Christine Stadelmann, Cristina Granziera

**Affiliations:** 1 Research Center for Clinical Neuroimmunology and Neuroscience Basel (RC2NB), University of Basel, Basel, Switzerland; 2 Translational Imaging in Neurology Basel, Department of Biomedical Engineering, Faculty of Medicine, University of Basel, Allschwil, Switzerland; 3 Department of Neurology, University Hospital Basel, Basel, Switzerland; 4 Department of Neuropathology, University Medical Center Göttingen, Göttingen, Germany

**Keywords:** Bielschowsky, Axon, Segmentation, Quantification, Attention U-net, Deep-learning

## Abstract

Reliable automatic quantification of axon density in histologic sections is important for both research and clinical neuropathology applications - however, it remains a challenge for silver-impregnation stains, such as Bielschowsky. Classical color-deconvolution approaches perform poorly on silver stains, and manual annotation of densely packed axons is time-consuming and subject to inter-rater variability. We developed an automated axon segmentation pipeline in Bielschowsky silver-stained histological brain sections based on an attention U-Net architecture with attention-gated skip connections, trained using Focal Tversky Loss to handle class imbalance and thin structure recovery. Ground truth was manually annotated on 33 image tiles (covering over 25 million pixels) derived from 26 whole-slide images from varying brain regions of four multiple sclerosis patients. Slides were prepared by different laboratory technicians at different time points to capture realistic staining variability. Model performance was evaluated on a held-out test set of unseen tiles. On the test set (eight held-out tiles) the model achieved a mean pixel-wise F1/Dice of 0.717 and an intersection over union (IoU) of 0.584. A dedicated inter-rater experiment on two representative tiles yielded rater-to-rater F1 scores of 0.637 and 0.671, while model-to-rater agreement (F1: 0.681–0.736) met or exceeded that human ceiling. Generated whole-slide density and orientation visualizations accurately reflected regional axon distributions and provided quantitative readouts suitable for downstream analysis. Our attention U-Net provides an open-source solution for axon segmentation from Bielschowsky-stained sections, reducing manual effort and enabling reproducible, slide-level quantitative metrics. This tool can facilitate studies of axonal pathology across research and clinical settings.

**Supplementary Information:**

The online version contains supplementary material available at https://doi.org/10.1007/s12021-026-09815-z.

## Introduction

Precise, reproducible quantification of axonal density is essential across neuroscience and neuropathology applications because axons constitute a major element of structural integrity and functional capacity of neuronal tissue (Kevenaar & Hoogenraad, 2015; Stadelmann et al., 2019). Quantitative measures of axonal loss or remodeling are central to the study of disease mechanisms combining histopathology and imaging (Gkotsoulias et al., 2025) and to the objective assessment of progression and therapeutic effects in conditions such as multiple sclerosis (MS) and other neurodegenerative disorders.

Silver impregnation methods - most commonly the Bielschowsky silver stain - remain widely used to visualize axons and neurofibrils in clinical and research histology (Bielschowsky, 1902; Nestor, 2008). However, converting silver-stained images into reliable quantitative maps remains challenging. Manual counting and stereological sampling are labor-intensive and scale poorly, while surrogate measures such as optical density and line-intersection counts are confounded by staining and sampling choices: optical density depends on staining quality and also captures non-axonal Bielschowsky-stained tissue, while line-intersection counts vary with arbitrary line placement and local fiber orientation across large tiles or whole-slide images (Fathy et al., 2022). In practice, these limitations reduce throughput and can introduce systematic bias that complicates comparisons across cohorts and studies.

Recent advances in convolutional neural networks have produced robust tools for axon and myelin segmentation in a variety of imaging modalities - for example AxonDeepSeg (Zaimi et al., 2018) and DeepACSON (Abdollahzadeh et al., 2021) - demonstrating that deep learning can deliver accurate, high-throughput morphometric analyses of neural tissue. Nevertheless, most existing deep learning pipelines were developed and validated on modalities such as electron microscopy, immunofluorescence, or myelin-specific stains, and to our knowledge no models optimized for brightfield silver-stained histology such as Bielschowsky exist. This modality is widely used in research and the clinical context, but presents distinct challenges (heterogeneous contrast, strong background staining) that can degrade the performance of models trained on other modalities.

Here we present an attention U-Net-based segmentation model (Ronneberger et al., 2015; Oktay et al., 2018) trained on manual annotations of axons and neurites sampled from multiple brain regions and from demyelinated lesions. The model produces dense, tile-level and whole-slide axonal segmentation maps that enable automated, reproducible axon density estimation and permit downstream morphometric analyses (e.g., fiber density maps and fiber-orientation/dispersion metrics). Compared with manual stereology or intensity-based surrogates, our approach increases throughput, removes arbitrary sampling decisions, and yields spatially resolved, directly interpretable axon maps that facilitate within-slide and between-cohort comparisons. We also provide a self-contained software package for straightforward deployment on tiled images and whole-slide scans, enabling integration into routine pathology-informatics workflows.

### Contributions

1. A robust attention U-Net trained on manually segmented Bielschowsky silver-stained histology, validated across multiple tissue classes (white matter, gray matter, demyelinated lesions, and background).
2. Demonstration of downstream utility: automated axon density maps and fiber-orientation metrics derived from segmentations.
3. A ready-to-deploy software application for tile-level and whole-slide processing to enable reproducible, high-throughput axon quantification.

## Methods

### Tissue Preparation and Histological Processing

The tissue samples used in this study were dissected from four brains from patients with MS, provided by the German MS Brain Bank of the Competence Network Multiple Sclerosis. This study was approved by the ethical review committee of the University Medical Center Göttingen. Representative samples of gray matter, white matter, and MS-lesion areas were manually chosen in each tissue sample. From within these, tiles sized 1024² pixels (pixel size 0.273 μm) were selected, and contrast enhancement was performed using Contrast Limited Adaptive Histogram Equalization (CLAHE; Zuiderveld, 1994) (clip limit: 4.0, grid size: 8 pixels). Manual ground truth segmentation of axons and dendrites on the resulting images was performed by two medical doctors. The two raters divided the annotation workload, so that each tile was segmented once by a single rater rather than by both (see Sect. 2.4). Cell bodies of all cell types were omitted from the segmentation. The same process was used for the validation and test sets (512² pixels). The reduced tile size was chosen as a practical tradeoff, balancing segmentation workload and coverage of tissue variability across the dataset. We deliberately selected tiles from diverse anatomical regions, tissue types, and WSIs to maximize biological and technical heterogeneity. A standardized 256 × 256 patch extraction ensures that smaller validation/test tiles do not introduce distributional shift and that the model observes identical spatial contexts regardless of source tile size. The finished ground truth masks were binary PNG images with values 0 for background and 255 for axons.

### Network Architecture

We developed an automated segmentation pipeline using an Attention U-Net architecture for binary classification of axonal structures in Bielschowsky-stained tissue sections. The network architecture followes the encoder-decoder structure originally proposed by Ronneberger et al. (2015) and was enhanced with attention gates as described by Oktay et al. (2018) to selectively emphasize task-relevant features while suppressing background information.

The encoder consisted of four hierarchical levels with progressive channel expansion (32, 64, 128, 256 channels) and spatial downsampling via $\:2\times\:2$ max pooling operations. Each encoder level comprised two sequential $\:3\times\:3$ convolutions, each followed by batch normalization and rectified linear unit (ReLU) activation. Dropout regularization (* p=0.3*) was applied at encoder levels 2–4 to prevent overfitting. The bottleneck layer expanded to 512 channels at an $\:8\times\:8$ spatial resolution.

The decoder mirrored the encoder structure with four upsampling levels, utilizing $\:2\times\:2$ transposed convolutions with stride 2. At each decoder level, attention gates modulated the corresponding encoder features before concatenation with the upsampled decoder features. The attention mechanism computed spatial attention coefficients $\:\alpha\:$ using the formulation:

$$\:\alpha\:\left(g,x\right)=\sigma\:\left({\psi\:}^{\top\:}\cdot\:\mathrm{ReLU}\left({W}_{g}\cdot\:g+{W}_{x}\cdot\:x+b\right)\right)$$

where * g* represents the gating signal from the decoder, * x* denotes the skip-connection features from the encoder, $\:{W}_{g}$ and $\:{W}_{x}$ are $\:1\times\:1$ convolutions for channel reduction, $\:\psi\:$ is a $\:1\times\:1$ convolution to a single channel, and $\:\sigma\:$ is the sigmoid activation function. The attention-weighted features were then computed as the element-wise product $\:x\odot\:\alpha\:$.

The final output layer consisted of a $\:1\times\:1$ convolution producing single-channel logits for binary segmentation. The complete network contained approximately 7.85 million trainable parameters with input dimensions of $\:256\times\:256\times\:3$ pixels (RGB) and output dimensions of $\:256\times\:256\times\:1$ pixel (binary mask).

### Loss Function and Optimization

To address the severe class imbalance inherent in axon segmentation (approximately 30% foreground vs. 70% background pixels), we employed Focal Tversky Loss (Abraham & Khan, 2018), which combines asymmetric penalty weighting with hard-example mining. The Focal Tversky Loss is defined as:

$$\:{L}_{FTL}={\left(1-TI\right)}^{\gamma\:}$$

where TI represents the Tversky Index (Tversky, 1977):

$$\:TI=\frac{TP}{TP+\alpha\:\cdot\:FP+\beta\:\cdot\:FN+\epsilon\:}$$

with TP, FP, and FN denoting true positives, false positives, and false negatives, respectively. We set $\:\alpha\:=0.4$ (false positive weight), $\:\beta\:=0.6$ (false negative weight), $\:\gamma\:=2.0$ (focusing parameter), and $\:\epsilon\:=1.0$ (smoothing constant). The asymmetric weighting ($\:\alpha\:<\beta\:$) prioritized recall over precision to minimize missed axon pixels, while the quadratic focusing parameter ($\:\gamma\:=2.0$) up-weighted difficult examples, directing learning toward challenging boundary regions. The choice of β = 0.6 is empirically supported by a post-hoc parameter sweep (Sect. 3.6).

The network was optimized using AdamW (Loshchilov & Hutter, 2017) with the hyperparameter settings used in the released training configuration. Learning rate scheduling employed ReduceLROnPlateau with a reduction factor of 0.5 and patience of 5 epochs. Gradient clipping with a maximum norm of 1.0 was applied to stabilize training. Training continued for a maximum of 100 epochs with early stopping (patience: 5 epochs) based on validation loss.

### Dataset Composition and Annotation Strategy

Manual annotation of axonal structures in Bielschowsky-stained tissue is extremely time-intensive, necessitating a pragmatic approach to dataset assembly. Two trained medical doctors annotated 33 tissue tiles from 26 distinct whole-slide images (WSIs) across four human brains (brains 1, 2, 3, and 4), capturing white matter (WM), gray matter (GM), background (BG), and demyelinated lesions (L) across multiple anatomical regions.

To make this labor-intensive task feasible, the two raters divided the annotation work rather than each annotating every tile: for model training and evaluation, every tile carried a single annotation from one rater, so the model never received two conflicting labels for the same image and no label fusion was performed. Before annotating, both raters agreed on a common segmentation protocol by jointly performing segmentations on several shared example tiles. The segmentations was mainly based on two rules: (i) segmenting only thin, dark silver-impregnated axonal fibers, excluding cell somata, and, (ii) where an axon passed through a soma, tracing it continuously through the soma rather than interrupting it. The first rater annotated all validation and test tiles and 13 of the 17 training tiles, while the second rater annotated the remaining 4 training tiles. The model was therefore trained and evaluated against a single, consistent annotation standard (all test tiles originate from the first rater) rather than a fusion of two rater styles.

WSI identifiers follow the format *brain_block* (e.g., 1_1, 1_2 represent different tissue sections from brain 1). The dataset was strategically partitioned to balance regional representation, maximize test set independence, and ensure consistent tile dimensions within validation and test sets:

- **Training set**: 17 tiles from 12 WSIs across 3 brains (1, 2, 3).
- **Validation set**: 8 tiles from 8 WSIs across 2 brains (3, 4).
- **Test set**: 8 tiles from 8 WSIs across 4 brains (1, 2, 3, 4).

The dataset composition is summarized in Table 1. Critically, 7 of 8 test WSIs (87.5%) were completely independent, never appearing in training or validation sets. Only one test WSI (2_3) was shared with the training set, but tiles represented spatially distinct tissue regions (WM in test set vs. GM in training). Similarly, only one WSI (3_13) contributed tiles to both training and validation sets, again with spatially non-overlapping regions (BG in validation set vs. GM in training). No WSIs were shared across all three data splits.

**Table 1:** Dataset composition by data split, WSI count, and anatomical region

Split	Tiles (*n*)	WSIs	Brains	Tile size(s) (px)	Regional distribution
Training	17	12	1, 2, 3	16 tiles @ 1024 × 1024 1 tile @ 2236 × 1866	WM: 5, GM: 5, Lesion: 4, BG: 3
Validation	8	8	3, 4	8 tiles @ 512 × 512	WM: 3, GM: 2, Lesion: 2, BG: 1
Test	8	8	1, 2, 3, 4	8 tiles @ 512 × 512	WM: 3, GM: 3, Lesion: 1, BG: 1

*WM* white matter, *GM* gray matter, *BG* background tissue

Regional composition was balanced across all sets to ensure model exposure to diverse tissue types during training and robust evaluation across anatomical contexts. Training tiles comprised 5 WM, 5 GM, 4 Lesion, and 3 BG regions. Validation and test sets maintained similar regional distributions (WM: 3, GM: 2–3, Lesion: 1–2, BG: 1 per set), enabling assessment of generalization across tissue categories.

#### Detailed WSI Assignments

*Training WSIs* *(12)*: 1_1 (3 tiles), 1_4^†^ (2 tiles), 2_1 (2 tiles), 2_2 (1 tile), 2_3^‡^ (1 tile), 3_1 (1 tile), 3_5^†^ (2 tiles), 3_6 (1 tile), 3_8 (1 tile), 3_12 (1 tile), 3_13^†^ (1 tile), 3_15 (1 tile).

*Validation WSIs* *(8)*: 3_2^*^, 3_3^*^, 3_9^*^, 3_10^*^, 3_11^*^, 3_13^†^, 3_16^*^, 4_3^*^ (all 1 tile each).

*Test WSIs (8)*: 1_2^*^, 1_3^*^, 2_3^‡^, 3_4^*^, 3_7^*^, 3_14^*^, 4_1^*^, 4_2^*^ (all 1 tile each).

^*^ WSI unique to this split. ^†^ WSI shared between training and validation (3_13) or within training only (1_4, 3_5 multi-tile). ^‡^ WSI shared between training and test. Shared WSI always represent spatially distinct regions.

### Patch Extraction Strategy

All tiles, regardless of original dimensions, were subdivided into standardized $\:256\times\:256$ pixel patches, ensuring consistent spatial context for the model across all data splits. Patch extraction employed two complementary strategies: Systematic grid extraction with a 256-pixel stride ensuring complete tile coverage and uniform random sampling introducing positional variability.

The final patch-level dataset comprised:

- **Training**: 17 tiles $\:\to\:$ 86,330 patches after 5-fold augmentation.
- **Validation**: 8 tiles $\:\to\:$ 2,032 patches (no augmentation).
- **Test**: 8 tiles $\:\to\:$ 2,032 patches (no augmentation).

#### Data Leakage Prevention

To prevent data leakage through spatial autocorrelation, all patches extracted from a given source tile were assigned exclusively to a single data split. Adjacent tissue regions within the same tile exhibit structural similarity due to local cytoarchitecture, myeloarchitecture and staining characteristics, making random patch-level redistribution across splits methodologically inappropriate. Where WSIs contributed tiles to multiple data splits, tiles were spatially non-overlapping and sampled from distinct anatomical locations.

The high degree of test set independence (7/8 WSIs completely independent, including 2 WSIs from brain 4 never observed during training) enables robust assessment of model generalization. These design constraints rendered traditional * k*-fold cross-validation infeasible. Instead, we employed leave-one-WSI-out (LOWO) analysis on the training set to assess model robustness to exclusion of individual training WSIs.

### Data Augmentation

Data augmentation was applied exclusively to the training set to simulate biological and technical variability, with five versions generated per original patch (1 original plus 4 augmented versions). Augmentation operations were applied using the Albumentations library (Buslaev et al., 2020). Spatial transformations were applied synchronously to images and masks, whereas intensity transformations were applied to images only.

### Performance Evaluation

Model performance was assessed using multiple metrics computed on binarized predictions (threshold: 0.5). Primary metrics included the Dice coefficient (F1-score) and Intersection over Union (IoU, Jaccard Index). Secondary metrics included precision and recall. The Dice coefficient was calculated as:

$$\:\mathrm{Dice}=\frac{2\left|X\cap\:Y\right|}{\left|X\right|+\left|Y\right|+{\upepsilon\:}}$$

and IoU as:

$$\:\mathrm{IoU}=\frac{\left|X\cap\:Y\right|}{\left|X\right|+\left|Y\right|-\left|X\cap\:Y\right|+{\upepsilon\:}}$$

where * X* and * Y* represent predicted and ground truth masks, respectively, and $\:\epsilon\:={10}^{-6}$ prevents division by zero.

#### Interrater Agreement Evaluation

To directly quantify annotation subjectivity, two independent medical-doctor raters each segmented the same two image tiles. The resulting masks were compared pairwise with each other and with the model prediction using Dice coefficient and IoU. These two tiles — the test tile 1_3 and the validation tile 4_3 — are the only tiles annotated independently by both raters. For model training and evaluation only the first rater’s annotation of each was used, consistent with the rest of the validation and test set, which the first rater also labelled. The second rater’s annotations were made solely for this inter-rater comparison and were not used during the model’s training and validation.

#### Leave-One-WSI-Out Analysis

In each of 12 folds, one unique WSI was held out from training and the remaining WSIs were used to train a separate model. Validation and test set were held constant. Performance was then evaluated on the fixed validation set using Dice coefficient, IoU, precision, and recall. Each LOWO model was trained from scratch using the same architecture, loss function, optimizer, and early stopping configuration as the full model. Online Resource 1 summarizes the training loss curves for all folds together with the corresponding validation performance.

#### Whole Slide Image Maps

Tile-level segmentation masks were aggregated to produce an axon density map (panel B), where pixel intensity reflects the fraction of foreground axon pixels per spatial bin. We chose a spatial bin of 70 × 70 μm to maintain anatomical detail while keeping the image size manageable. The spatial bin size can be easily adjusted in the software application. Axon orientation was quantified using a grid-based structural-tensor approach applied to the binary whole-slide axon segmentation mask. The mask was subdivided into a regular grid of non-overlapping square cells (again with a spatial bin of 70 × 70 μm), and for each cell the following procedure was performed. Image gradients were computed using the Scharr operator (Scharr, 2007). The outer-product structure tensor was then assembled per pixel as:

$$\:\mathbf{J}\:=\left(\begin{array}{cc}{g}_{x}^{2}&\:{g}_{x}\:{g}_{y}\\\:{g}_{x}\:{g}_{y}&\:{g}_{y}^{2}\end{array}\right)$$

and each component was smoothed with a Gaussian kernel of standard deviation σ = 2 pixels to integrate local neighborhood information. The cell-level tensor $\:\stackrel{-}{\mathbf{J}}$ was obtained by averaging $\:\mathbf{J}$ over all axon-positive pixels within the cell. Eigenanalysis of $\:\mathbf{J}$ yielded the dominant $\:{\lambda\:}_{1}$and secondary $\:{\lambda\:}_{2}$ eigenvalues, with corresponding eigenvectors defining the primary orientation $\:{\theta\:}_{1}\:$and perpendicular secondary orientation $\:{\theta\:}_{2}$, both expressed in the range [0°, 180) via

$$\:\theta\:=atan2\left({v}_{y},{v}_{x}\right)\:mod{180}^{\circ\:}$$

Alignment strength per cell was quantified as the 2D fractional anisotropy (FA)-analog coherency:

$$\:Coherency=\frac{\left|{\lambda\:}_{1}-{\lambda\:}_{2}\right|}{{\lambda\:}_{1}+{\lambda\:}_{2}}$$

ranging from 0 (isotropic, no preferred direction) to 1 (perfectly aligned). Final output maps consisted of an axon density map, a coherence map and an HSV-encoded summary image in which hue encodes $\:{\theta\:}_{1}$, saturation encodes coherency, and value indicates axon presence (set to 1 if axons are present within the grid cell and to 0 in empty cells).

### Implementation

The segmentation pipeline was implemented using PyTorch 2.x (Paszke et al., 2019) with PyTorch Lightning for training orchestration. Data augmentation utilized the Albumentations library (Buslaev et al., 2020), and experiment tracking was performed using Weights & Biases (n.d.). Training was conducted on an NVIDIA RTX 2000 Ada GPU (8 GB VRAM) with batch size 16. Reproducibility was ensured by setting a global random seed (42) and enabling deterministic algorithms. Model selection was based on minimum validation loss, with the top three models and the most recent checkpoint preserved.

### Baseline Comparison (nnU-Net)

As an external baseline we trained nnU-Net v2 (Isensee et al., 2021), a widely used self-configuring segmentation framework, on the identical data. To keep the comparison controlled, nnU-Net received the same 256 × 256 patches and the same data partition as our model: the 17,328 un-augmented training patches and 2,032 validation patches were supplied with our exact train/validation split imposed as a custom single fold, and the 2,032 test patches were held out. The 2D configuration was trained with nnU-Net’s default self-configuring pipeline (automated planner, 256 × 256 patch size matching our field of view, 1000 epochs, the framework’s own on-the-fly data augmentation), and its standard best-validation checkpoint (selected on validation pseudo-Dice) was used for inference. No test-time post-processing was applied, matching our model. nnU-Net predictions were binarized at 0.5 and evaluated with the identical stratified per-tile protocol (Sect. 2.7), so the resulting metrics are directly comparable to those of our Attention U-Net. For the qualitative whole-tile comparison, each model was additionally applied to the full 512 × 512 test tiles (nnU-Net through its standard sliding-window inference).

## Results

### Training Performance

The full model (7.9 million trainable parameters) was trained on a total of 86,330 augmented patches (5,415 gradient steps per epoch, batch size 16) for a maximum of 100 epochs with early stopping monitored on validation loss (patience: 5 epochs, minimum improvement threshold: $\:{10}^{-4}$).

**Fig. 1:**
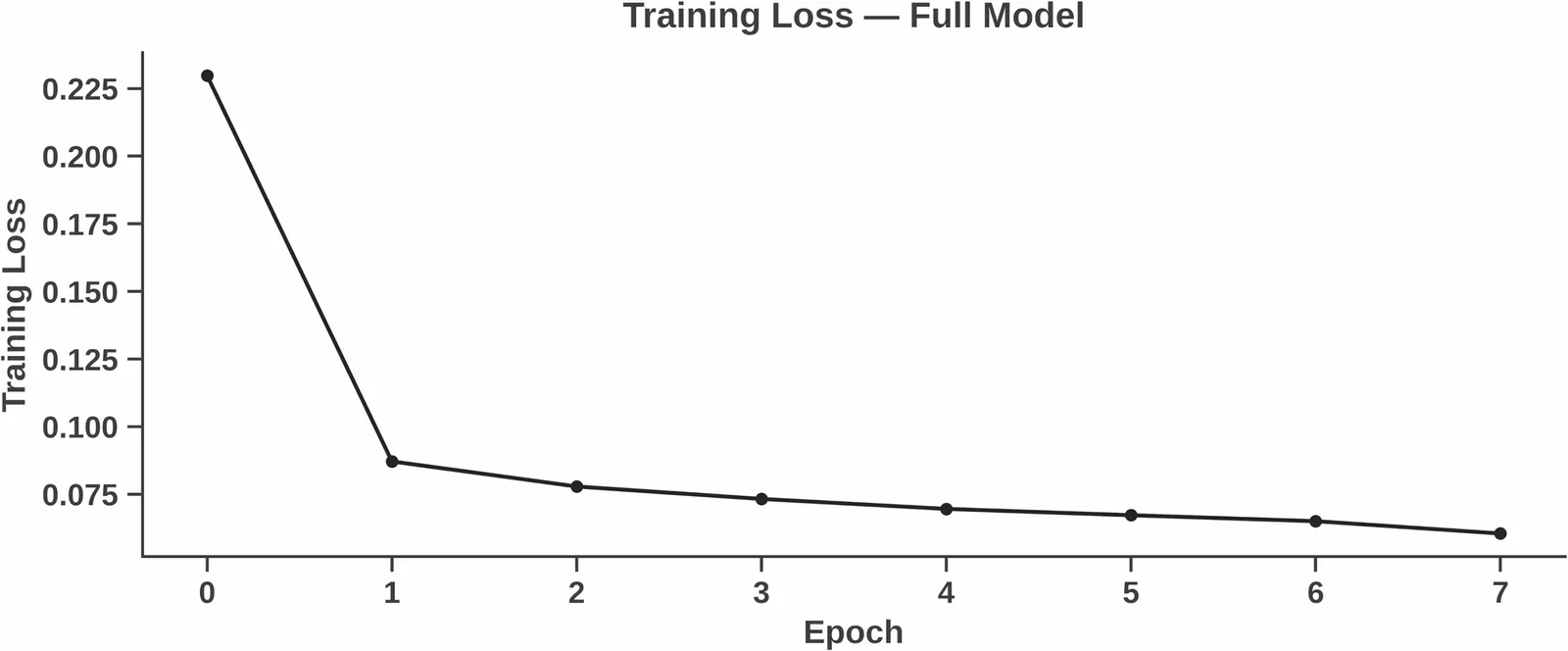
Training loss (Focal Tversky) across epochs for the full model

Training converged rapidly. The Focal Tversky Loss decreased steeply during the first two epochs and reached its minimum on the validation set at epoch 2 (val/loss * 0.0528*; Fig. 1). Epochs 3 through 7 showed no further improvement in validation loss, triggering early stopping after a total of 8 epochs (ca. 5.5 h on an NVIDIA RTX 2000 Ada). The best checkpoint (epoch 2) was restored for all downstream evaluations.

**Fig. 2:**
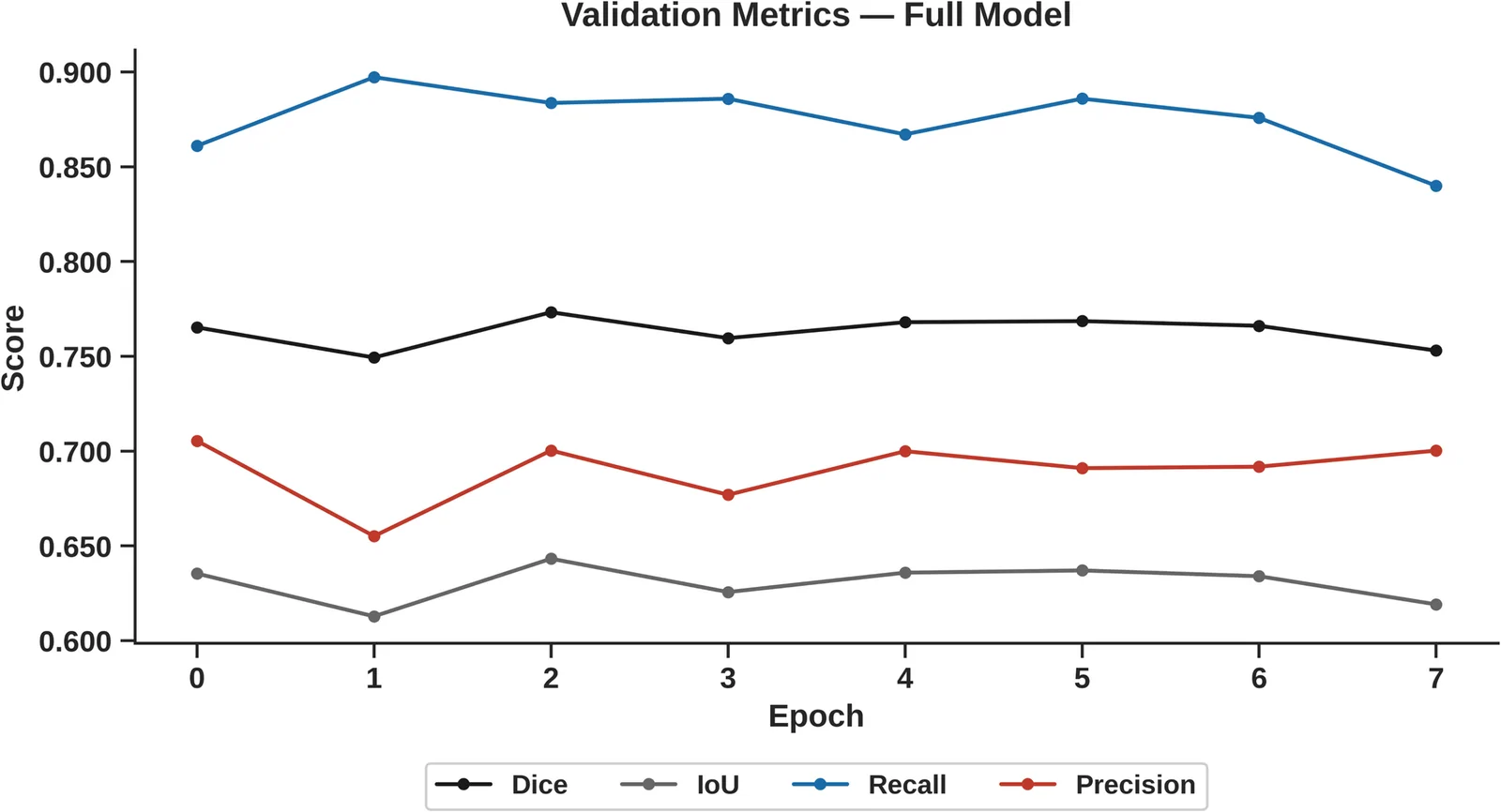
Validation metric curves across training epochs. The best checkpoint was selected at epoch 2 (val/loss = 0.0528). Early stopping terminated training after epoch 7 with no further improvement. Recall exceeded precision throughout training, consistent with the recall-prioritizing loss design

All validation metrics (Precision, Recall, F1, IoU) tracked the validation loss improvement closely, with their best values reached at or near the same checkpoint (Fig. 2). The recall ($\:\approx\:0.75$ at best checkpoint) consistently exceeded precision ($\:\approx\:0.68$), reflecting the asymmetric Focal Tversky loss design which penalizes false negatives more heavily than false positives ($\:\beta\:=0.6>\alpha\:=0.4$).

### Ablation Study: Loss Weighting and Attention Gates

To empirically isolate the two important design choices — the Focal Tversky weighting and the attention-gated skip connections — we performed two ablations under the same held-out test set and stratified per-tile evaluation used above (8 tiles, threshold 0.5; Supplementary Results 1). All other settings (architecture, optimizer, augmentation, and the focusing parameter γ = 2.0) were held fixed.

#### Loss Weighting

We swept the Tversky weightings along α + β = 1 from β = 0.1 to β = 0.9 (Supplementary Tables 1 and Supplementary Fig. 3). Test Dice peaked at β = 0.6 (Dice = 0.715), the value used in the main model, but the top of the curve was a broad plateau: settings from β = 0.4 to β = 0.8 all scored within approximately 0.01 Dice of the maximum, a difference smaller than the tile-to-tile standard deviation. As expected, precision decreased and recall increased monotonically with β. We therefore regard β = 0.6 as the highest-scoring setting; our selection was additionally motivated by a deliberate, task-motivated preference for recall over precision, since for axon-density quantification a missed axon was considered more detrimental than a slight over-segmentation.

#### Attention Gates

At the selected loss configuration (α = 0.4, β = 0.6) we compared the network with and without the attention-gated skip connections, training three independent seeds of each (Supplementary Fig. 4). Enabling the attention gates improved mean test Dice from 0.699 to 0.715 (mean ΔDice = + 0.017), and the improvement was consistent across all three seeds and all five metrics (Dice, IoU, precision, recall, F1), at a cost of only about 0.09 M additional parameters (approximately 1% of the model). The effect is thus small but consistent and essentially free in model size, and we observed no downside to including the gates.

### Test Set Performance

**Fig. 3:**
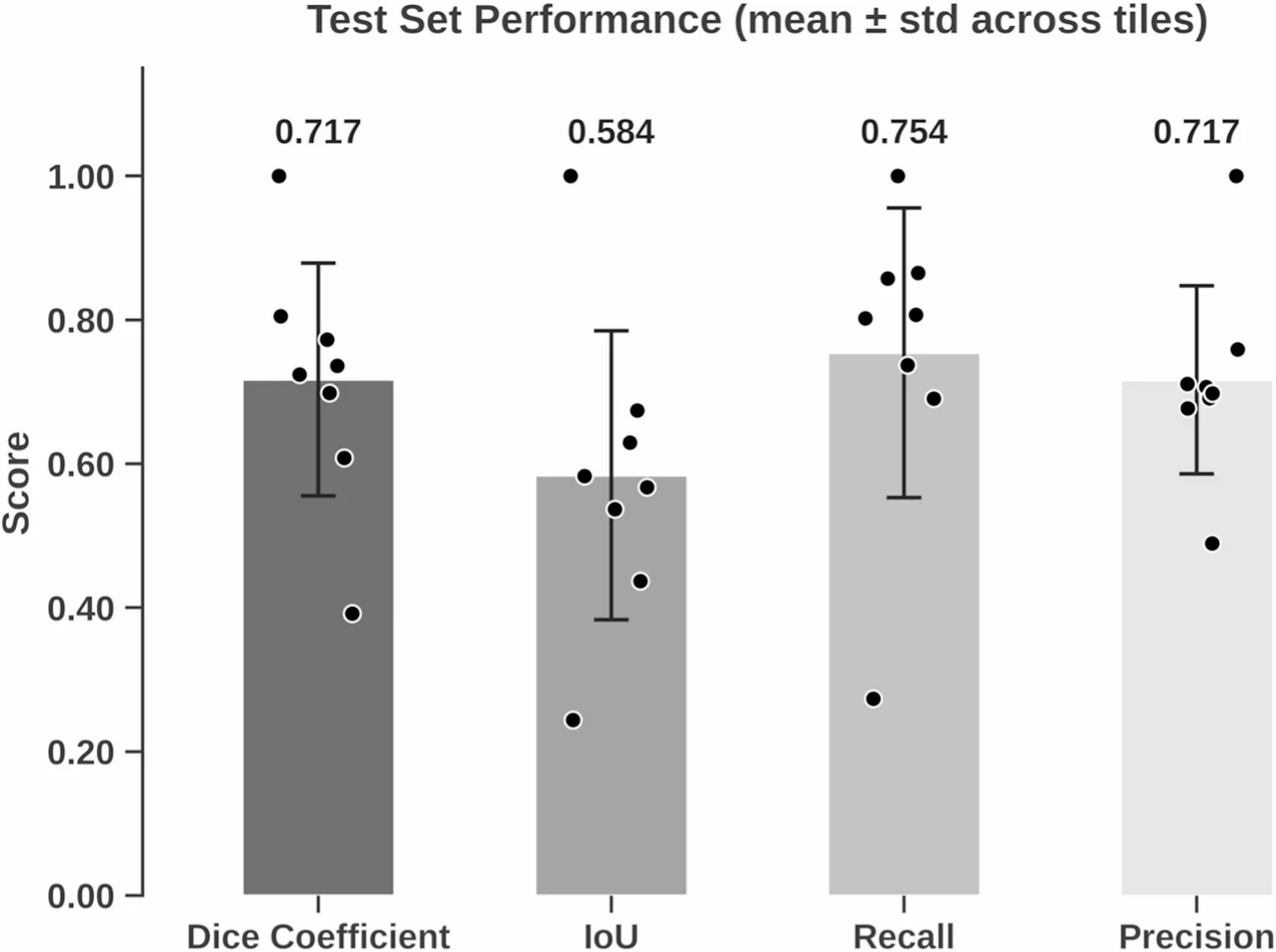
Aggregated test set performance across the 8 test tiles for F1, IoU, recall, and precision. T-bars indicate the standard deviation across the 8 test tiles. Overlaid points show the eight individual per-tile values for each metric

On the held-out test set the model achieved a mean pixel-wise F1/Dice of $\:0.717\pm\:0.162$ and an IoU of $\:0.584\pm\:0.201$ (mean $\:\pm\:$ SD across the 8 test tiles; Table 2). Mean recall ($\:0.754\pm\:0.201$) exceeded mean precision ($\:0.717\pm\:0.131)$, consistent with the design intent of the Focal Tversky Loss to prioritize recall and minimize missed axon pixels (Fig. 3).

**Table 2:** Per-tile test set performance metrics. Tile IDs follow the convention *brain_block*. Best and worst Dice scores are bold. Mean ± SD is computed across 8 tiles (254 patches each)

Tile ID	IoU	Recall	Precision	Dice
1_1	0.537	0.691	0.707	0.699
1_3	0.567	0.737	0.711	0.724
2_3	0.674	0.857	0.759	0.805
3_4	1.000	1.000	1.000	**1.000**
3_7	0.437	0.802	0.490	0.608
3_14	0.244	0.274	0.691	**0.392**
4_1	0.630	0.865	0.698	0.773
4_2	0.583	0.807	0.677	0.736
Mean ± SD	0.584 ± 0.201	0.754 ± 0.201	0.717 ± 0.131	0.717 ± 0.162

Performance varied considerably across individual test tiles. The highest Dice score was achieved on tile 3_4 (Dice * 1.00*, IoU * 1.00*), while the lowest was observed on tile 3_14 (Dice * 0.392*, IoU * 0.244*). Note that tile 3_4 was a background tile, explaining the high evaluation performance. The low performance on tile 3_14 can be explained by the very low staining quality, making axon detection difficult also for human raters.

**Fig. 4:**
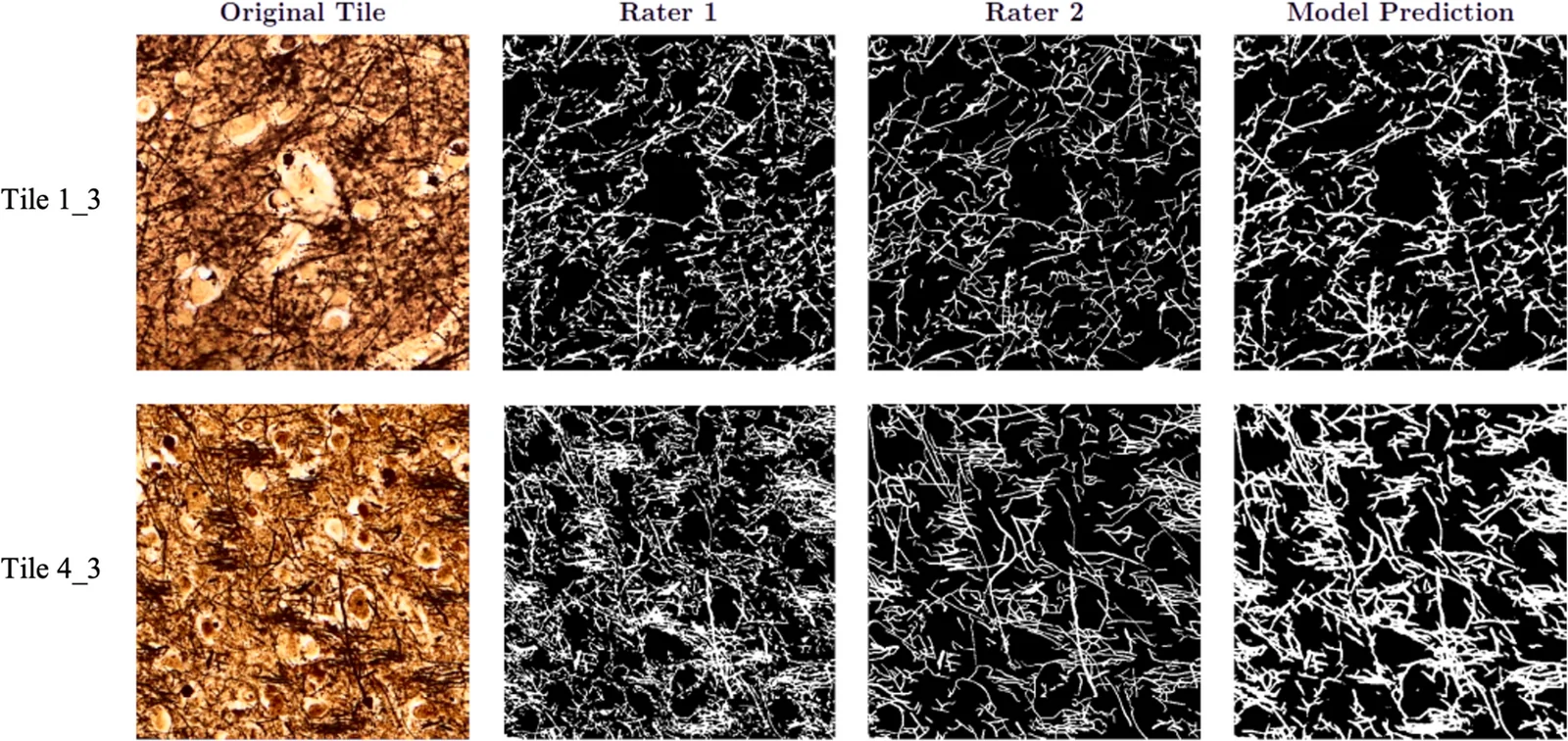
Inter-rater comparison of axon segmentation masks for two tiles. Row 1 shows tile 1_3 and row 2 shows tile 4_3. Columns from left to right show the original Bielschowsky-stained tissue patch, rater 1 annotation, rater 2 annotation, and model prediction. Visible differences between the two rater masks reflect the inherent subjectivity of the annotation task, particularly for fine dendrite branches, low-contrast fibres, and ambiguous background regions. White denotes axon foreground and black denotes background

Notably, the overall performance of * 0.717* Dice is encouraging in the context of the substantial inter-rater variability inherent in manual axon annotation (Table 3; Fig. 4), suggesting that the model approaches the practical upper bound of reproducible annotation quality.

**Table 3:** Pairwise agreement between rater annotations and model predictions for the two tiles used in the inter-rater variability analysis. The rater-to-rater row (shown in bold) represents the human agreement ceiling; model-to-rater rows show how the model compares relative to that ceiling. F1/Dice and IoU are computed on binarized masks (threshold = 0.5)

Tile ID	Comparison	Dice	IoU
1_3	**Rater 1 vs. Rater 2**	**0.637**	**0.467**
	Rater 1 vs. Model	0.736	0.582
	Rater 2 vs. Model	0.703	0.542
4_3	**Rater 1 vs. Rater 2**	**0.671**	**0.504**
	Rater 1 vs. Model	0.723	0.566
	Rater 2 vs. Model	0.681	0.516

### Inter-Rater Agreement

F1-scores between the two raters were * 0.637* for tile 1_3 and 0.671 for tile 4_3. The visual differences between the two rater masks (Fig. 4) further illustrate the reasons: both raters segment similarly the major axons, while thin branches and low-contrast fibers are demonstrating differences. Another valid point of difference is each rater’s perception of axonal width.

Critically, the model’s agreement with each individual rater (F1: 0.736 for rater 1 and 0.703 for rater 2 on tile 1_3; 0.723 for rater 1 and 0.681 for rater 2 on for tile 4_3) met or exceeded the rater-to-rater agreement on both tiles. These results demonstrate that the model’s predictions are as consistent with each human annotation as the two human annotators are with each other, confirming that the model operates within the intrinsic variability of the annotation task.

### Leave-One-WSI-Out Cross-Validation

**Fig. 5:**
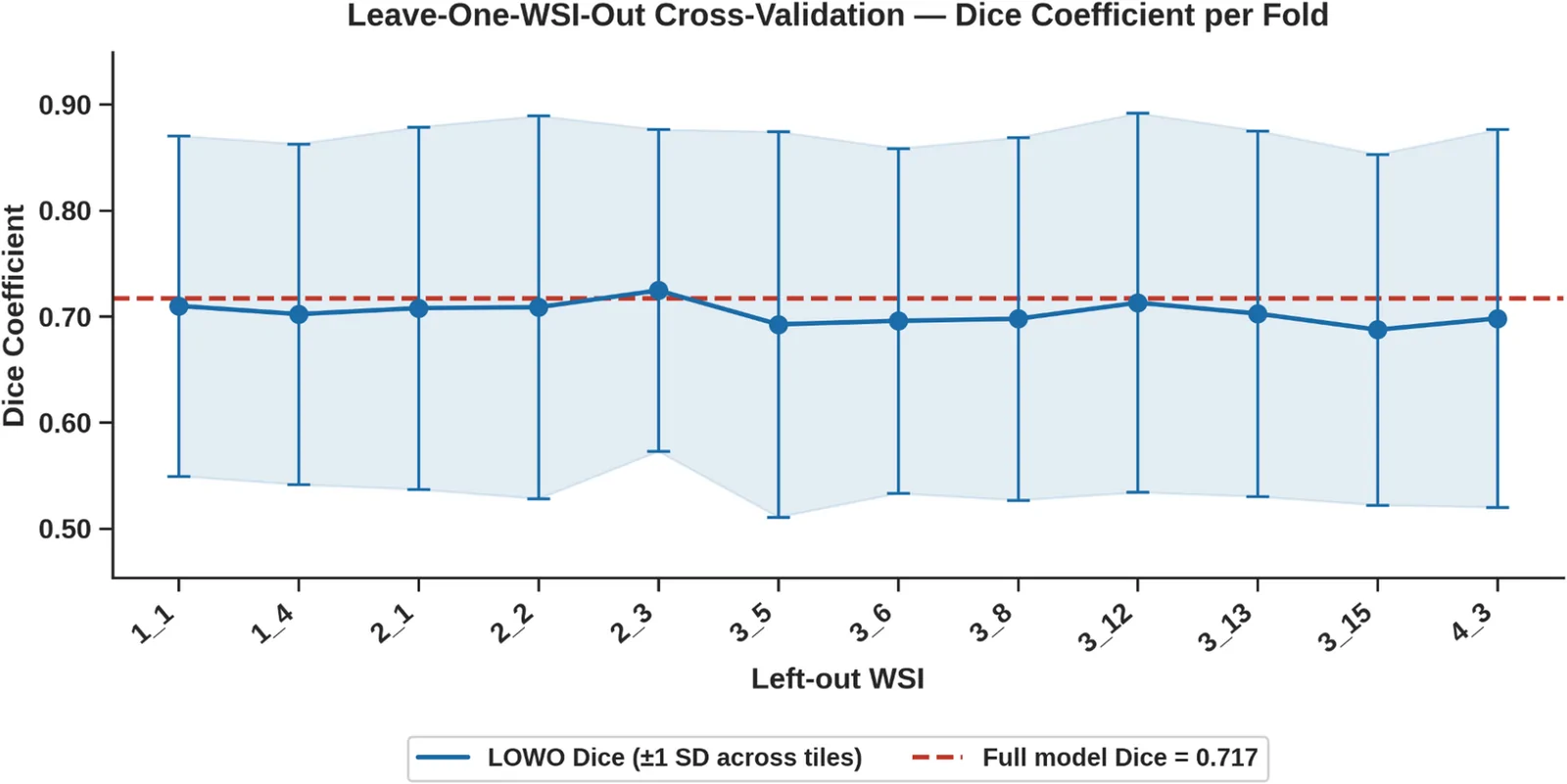
Dice score across 12 leave-one-WSI-out folds. Each point corresponds to the aggregated Dice score of one-fold in which the labeled WSI was excluded from the training set. T-bars indicate the standard deviation over the test set, and the dashed line indicates the full model mean Dice for comparison (0.717 ± 0.131)

LOWO models were evaluated on checkpoints at validation loss minima, which occurred between epoch 1 and epoch 10 across folds (median: epoch 4–5), again demonstrating rapid convergence. Across all 12 folds, the LOWO analysis yielded a mean Dice of $\:0.703\pm\:0.010$ (Table 4; Fig. 5), with limited inter-fold variability (range: 0.687–0.725). The low standard deviation suggests that no single training WSI is disproportionately critical to performance. The highest fold performance was achieved when WSI 2_3 was excluded (Dice * 0.725*), while the lowest was observed when WSI 3_15 was left out (Dice * 0.687*).

**Table 4:** Leave-One-WSI-Out (LOWO) cross-validation results across 12 training folds. Each row reports validation set performance when the indicated WSI is removed from training. Mean ± SD is computed across all 12 folds

Fold	Tile ID	IoU	Recall	Precision	Dice
0	1_1	0.575	0.762	0.680	0.710
1	1_4	0.566	0.745	0.688	0.702
2	2_1	0.575	0.782	0.678	0.708
3	2_2	0.578	0.736	0.712	0.709
4	2_3	0.591	0.802	0.680	0.725
5	3_5	0.560	0.823	0.623	0.693
6	3_6	0.559	0.762	0.662	0.696
7	3_8	0.562	0.727	0.707	0.698
8	3_12	0.583	0.772	0.696	0.713
9	3_13	0.569	0.765	0.677	0.703
10	3_15	0.549	0.708	0.689	0.687
11	4_3	0.565	0.737	0.691	0.698
Mean ± SD		0.569 ± 0.012	0.760 ± 0.032	0.682 ± 0.023	0.703 ± 0.010

### Qualitative Results

**Fig. 6:**
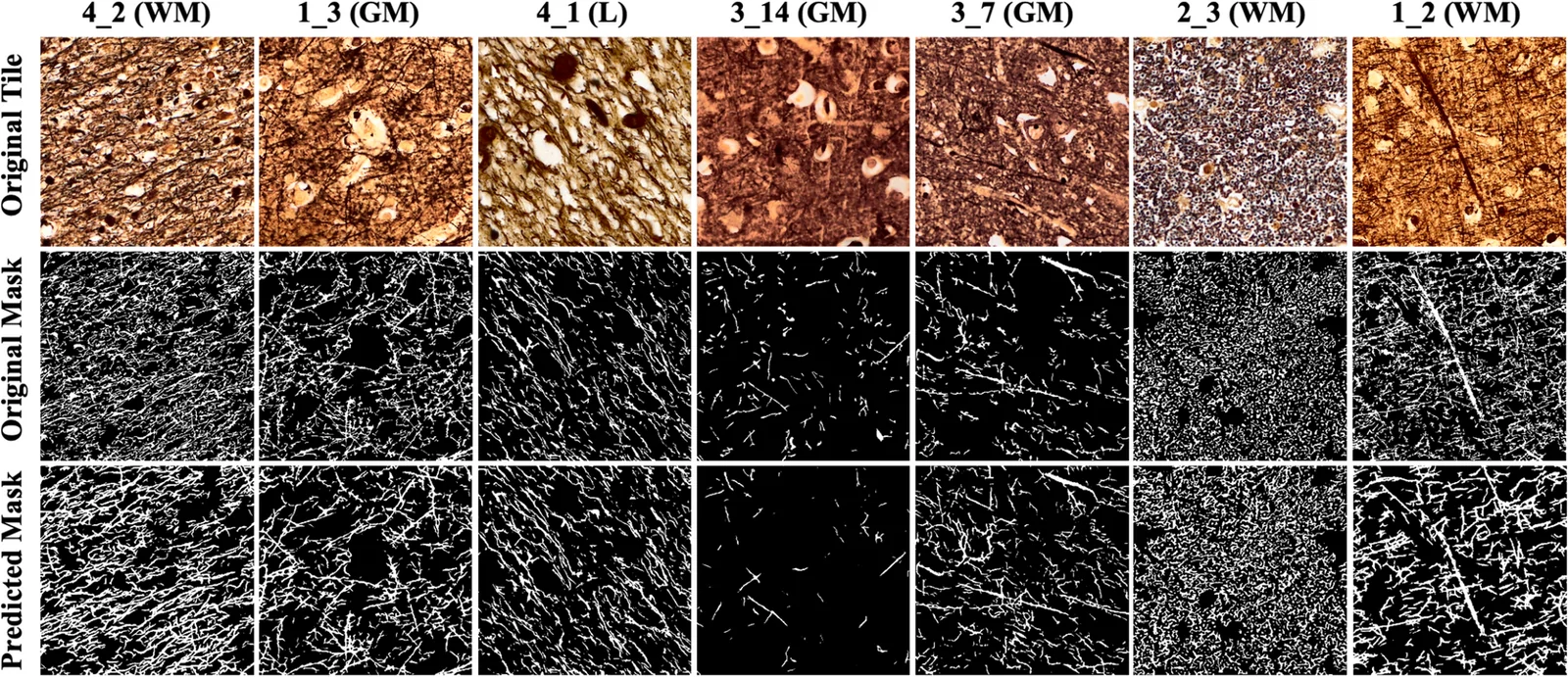
Qualitative test set results. Top row shows Bielschowsky-stained tissue patches from 7 test tiles. Middle row shows the manual annotation masks. Bottom row shows the corresponding predicted axon segmentation masks. Columns indicate tile ID and tissue type. White denotes axon foreground and black denotes background. The masks shown are the first rater’s annotations, i.e. those used for model evaluation. A second, independent annotation exists only for the inter-rater tile 1_3 (Sect. 2.7.1), so a uniform second-rater row for the test set cannot be shown

Figure 6 shows 7 test set tiles (the background tile 3_4 with Dice 1.000 is not shown) alongside their corresponding model-predicted axon segmentation masks. The model correctly recovered the densely packed axon network in white matter regions and successfully suppressed non-axonal structures such as cell bodies and background artefact. Prediction accuracy was reduced in regions with increased background staining and reduced axon density.

**Fig. 7:**
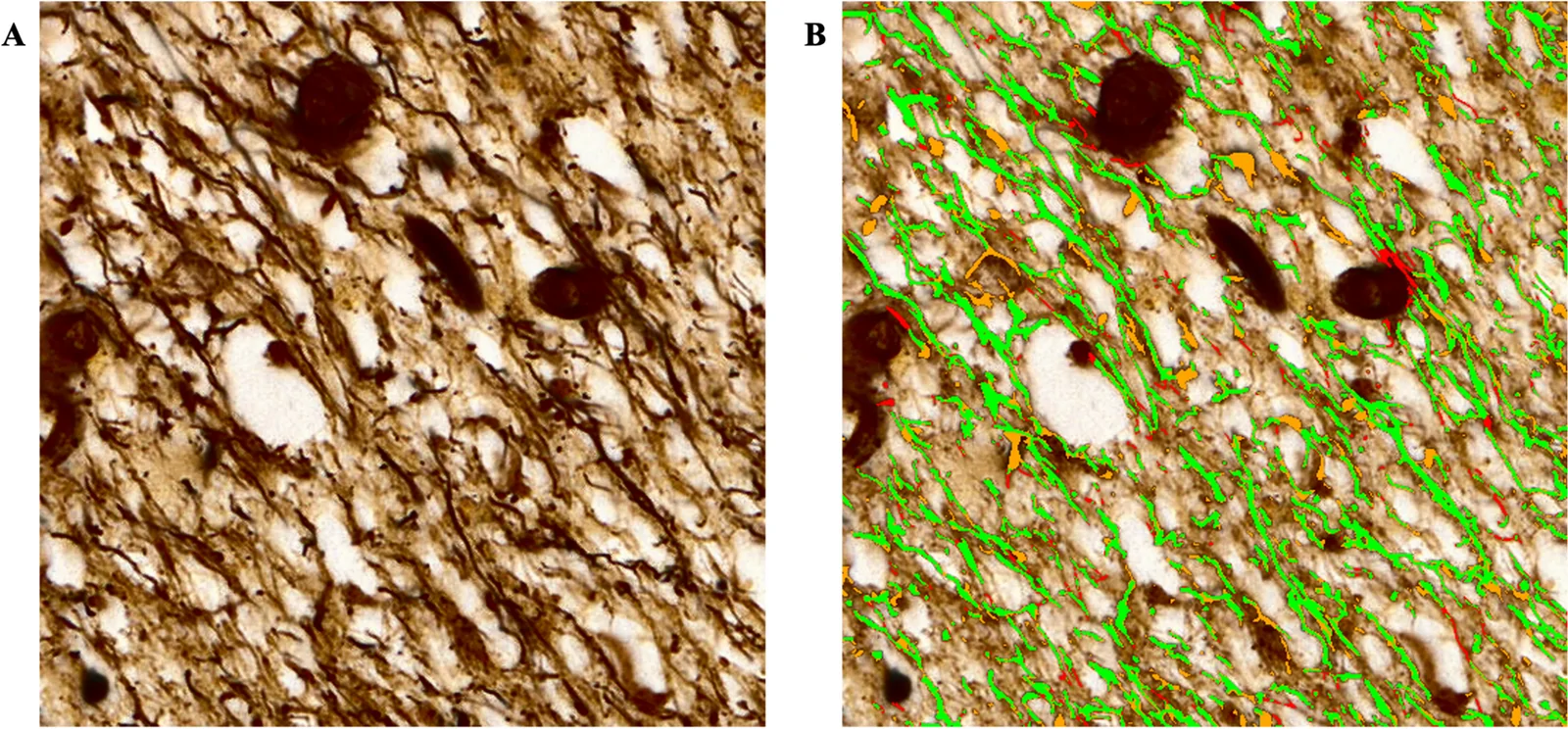
Qualitative evaluation for test tile 4_1 (Dice = 0.773, IoU = 0.630). (**A**) Original Bielschowsky-stained input patch. (**B**) Color-coded prediction overlay: green pixels indicate true positives (correctly segmented axons), red pixels indicate false negatives (missed axons), and orange pixels indicate false positives (over-segmentation)

Figure 7 shows a color-coded prediction overlay for tile 4_1, which achieved Dice * 0.773* on the test set. The overlay depicts true-positive (green), false-negatives (red), and false-positives (orange) detections, enabling direct visualization of prediction errors within the tissue context. While axons are delineated with high accuracy in general, fine dendrite structures are often false-negative. False-positives arise mainly in regions with ambiguous stain or due to background noise.

**Fig. 8:**
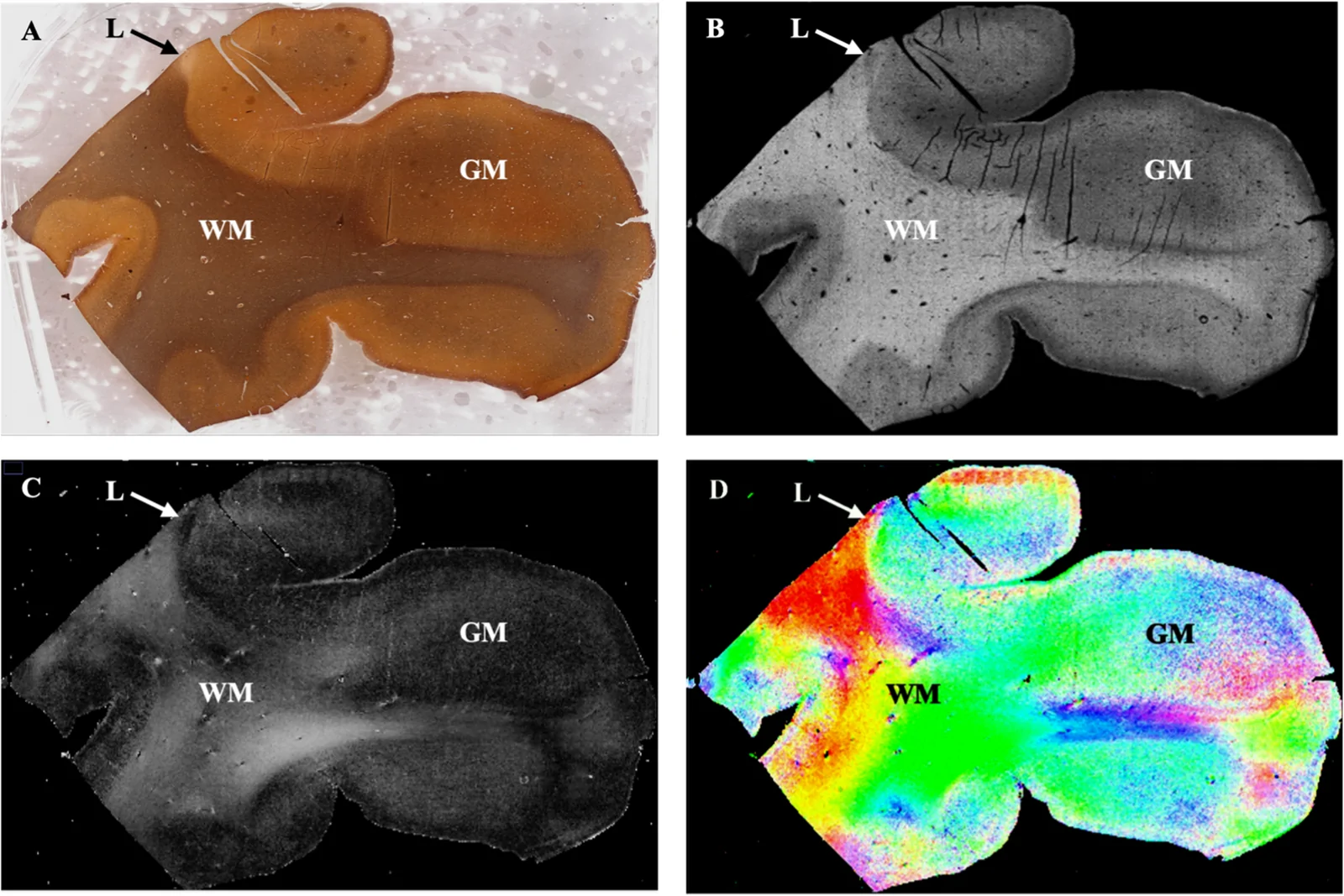
Whole-slide applicability demonstrated on tissue block 1_3. Panel (B-D) share the same spatial coordinate frame at 70 μm resolution. *L* lesion, *WM* white matter, *GM* gray matter. (**A**) Original Bielschowsky-stained whole-slide image (down sampled). (**B**) Axon density map derived from aggregated tile-level segmentation masks; intensity reflects the proportion of axon foreground pixels per spatial bin (brighter= higher density). White matter tracts appear uniformly bright; grey matter cortex and lesion area show reduced density. (**C**) Fiber coherency map from structural tensor analysis (brighter= higher directional alignment). Compact white matter tracts are highly coherent; grey matter, crossing-fiber zones, and lesion areas exhibit low coherency. (**D**) Dominant fiber orientation encoded by HSV color wheel (hue encodes direction ∈ [0°,180°); saturation encodes coherence). Smooth color transitions indicate coherent fiber bundles; color boundaries correspond to tract boundaries or crossing regions

To demonstrate applicability beyond isolated tiles, we applied the trained model to a complete Bielschowsky-stained whole-slide image (WSI 1_3) and derived three spatially resolved maps, downsampled to 70 μm resolution (Fig. 8). The resulting map captures the expected regional anatomy: white matter regions exhibit uniformly high axon density, whereas the cortical gray matter and the MS lesion area show reduced density, consistent with the known cytoarchitecture of the tissue.

The orientation map (panel D) reveals varying fiber direction domains that correspond to white matter tracts. The coherency map (panel C) quantifies the degree of directional alignment of local axonal architecture. Highly organized fiber bundles correspond to brighter regions, whereas crossing-fiber regions, cortex, and lesion areas exhibit lower coherency (darker regions).

### Baseline Comparison (nnU-Net)

Across all eight test tiles our Attention U-Net attained a higher mean Dice than nnU-Net (0.717 ± 0.162 vs. 0.555 ± 0.245; IoU 0.584 ± 0.201 vs. 0.417 ± 0.200; paired Wilcoxon signed-rank on per-tile Dice, *p* = 0.023, *n* = 8; Supplementary Results 2, Supplementary Table 2). This full-set gap was strongly influenced by the background tile 3_4, whose empty ground truth makes the pixel Dice degenerate: our model predicted no axons there (Dice 1.000), whereas a few false-positive pixels drove nnU-Net’s patch-pooled score to 0.000, even though its whole-tile prediction on this tile is likewise essentially empty (Supplementary Fig. 5). To report the comparison fairly, we therefore also evaluated the seven axon-bearing tiles, excluding this degenerate background tile. On these tiles the two models were closer, but our model remained ahead on Dice (0.677 ± 0.130 vs. 0.634 ± 0.135; *p* = 0.047, *n* = 7), with a complementary error profile: the Attention U-Net achieved higher recall (0.719 vs. 0.606) whereas nnU-Net was more precise (0.749 vs. 0.676). Per-tile metrics for both models are given in Supplementary Tables 3, and a whole-tile qualitative comparison in Supplementary Fig. 5. Notably, our model reached this performance with far fewer parameters (7.9 M vs. approximately 48 M for nnU-Net’s self-configured network).

## Discussion

We present an attention U-Net trained to segment axons in Bielschowsky silver-impregnated histological sections. The achieved mean pixel-wise F1/Dice score and Leave-one-WSI-out cross-validation on the held-out test set, spanning four brains, multiple tissue classes, and both normal and demyelinated tissue, demonstrated generalizable and robust model performance. Furthermore, we demonstrate that the binary segmentation output can be aggregated to full WSI-level maps of axon density, dominant fiber orientation, and local fiber coherency (Fig. 8), establishing a practical pipeline from raw silver-stained scan to spatially resolved quantitative readouts.

For thin, densely packed structures such as axons in silver-stained tissue, human-level agreement is itself limited. In our own annotation experiment on two representative tiles, rater-to-rater F1 was 0.637 and 0.671, whereas the model-to-rater agreements were 0.681–0.736 (Table 3), consistently at or above the human ceiling. Comparisons for similar tasks are rare in the literature. Golabchi et al. (2010) reported stereological inter-rater variability for axon counting in gray matter. Deng et al. (2021) reported a Dice coefficient of 0.78 between two raters for pixel-wise axon segmentation in paraphenylenediamine-stained optic nerve sections, and Kumar et al. (2020) reported an average aggregated Jaccard index of 0.653 in pixel-wise nuclei segmentation in hematoxylin-eosin sections. Our model showed a Dice score of 0.72, indicating that it operates within the range of inter-rater disagreement and suggesting that the principal remaining source of error is annotation ambiguity rather than model-induced limitations.

Two design choices were central to the model’s performance: attention-gated skip connections and the Focal Tversky Loss. The attention mechanism allows the decoder to selectively weight encoder features according to relevance at each spatial location, suppressing responses in background regions while preserving detail in axon-dense areas. This is particularly valuable in Bielschowsky-stained tissue, where heterogeneous background silver deposition can produce false-positive activation in a standard U-Net. The Focal Tversky Loss addresses the severe class imbalance inherent in axon segmentation (approximately 30% foreground pixels) by penalizing false negatives more heavily than false positives ($\:\beta\:=0.6>\alpha\:=0.4$) and applying a quadratic focusing factor ($\:\gamma\:=2.0$) that concentrates learning on difficult examples. The consequence is visible in the test set metrics: mean recall (* 0.754*) consistently exceeded mean precision (* 0.717*), indicating that the model errs toward detecting axons rather than missing them, which we consider a less detrimental error pattern for density quantification, where missed axons directly reduce density estimates. To empirically substantiate these two choices, we performed a Tversky-weighting sweep and an attention on/off comparison (Sect. 3.2). The sweep identified β = 0.6 as the highest-scoring weighting, situated at the top of a broad plateau, and our choice additionally reflects a task-motivated preference for recall over precision. The attention gates provided a small but consistent improvement across seeds at negligible parameter cost. We therefore regard both as sensible, empirically supported design choices.

Beyond tile-level evaluation, the WSI-level density, orientation, and coherency maps derived from the model output (Fig. 8) illustrate the practical utility of the pipeline for research and lab-routine applications. The density map recapitulates the expected gross anatomy of the tissue block: compact white matter with uniformly high axon density, a cortical gray matter ribbon with lower and more variable density, and visibly reduced density in MS-lesion areas. The structural tensor–derived orientation and coherency maps provide an orthogonal readout, where fiber coherency is increased in organized, highly-directional WM tracts and decreased at GM boundaries and lesion borders, consistent with the known disruption of myeloarchitecture in those regions.

Our work comes with some limitations. Despite training and test tiles were resected from different brains, different anatomical locations and were prepared at different time points by more than one technician, they originate from a single disease context (multiple sclerosis), and a single staining protocol was followed. This might constitute a limiting factor on generalization over different silver impregnation protocols. Expanding the training dataset to include additional brains, other disease contexts (e.g., Alzheimer’s disease, traumatic brain injury), and sections stained in different laboratories would improve generalization and allow a more rigorous evaluation of domain robustness.

Regarding the practical scope of the model, several boundaries should be kept in mind. All training data were digitized at a single resolution (0.273 μm per pixel); when we applied the trained model, without retraining, to Bielschowsky sections digitized at a coarser resolutions (0.35 μm per pixel and 0.70 μm per pixel), the segmentations remained visually convincing (Supplementary Results 3, Supplementary Fig. 6). We did not, however, annotate data at other resolutions and therefore cannot quantify performance there, and behavior under more pronounced changes in resolution is unknown. Because our training tiles deliberately include sections prepared by different laboratory technicians at different time points, and because we apply strong data augmentation, we expect the model to transfer reasonably to Bielschowsky material from other laboratories acquired at a comparable resolution; we have, however, no data from other laboratories to confirm this, and generalization to other silver-impregnation stains was not evaluated. Where such domain shifts prove too large, a foundation-model-based approach (e.g., fine-tuning a large pretrained segmentation model) is a promising future direction for broadening applicability.

The annotation set is relatively small (* n=33* tiles), which might constrain both the diversity of the training set and the statistical power of the evaluation. Additionally, the complexity of the annotation task (particularly for thin dendrite branches and regions of non-specific background staining) propagates directly into the reported performance metrics. Involvement of more primary annotators for the segmentations, would potentially provide a better-grounded ceiling for model performance and help disentangle model error from annotation noise. Because dense axon annotation is so labor-intensive, each tile was segmented only once, by a single rater (except the two tiles of the dedicated inter-rater experiment); we therefore lack repeated independent annotations of the modelling tiles, and the inter-rater ceiling was estimated from two tiles rather than the full set.

Despite strong mean performance, it is important to note that test Dice scores ranged widely from ~ 0.4 to 1 across the eight test tiles, reflecting the influence of staining quality and regional complexity on segmentation performance. Uniformly stained tissue regions with clearly distinguishable axonal structures achieved the highest performance metrics, whereas tiles with severe background stain, complex structural details and lacking contrast exhibited substantially lower segmentation accuracy. However, similar challenges also affect manual segmentation, where poor staining quality (e.g., non-specific silver precipitates, stain inhomogeneities, and intricate tissue morphology), likewise complicate accurate annotation. To our knowledge, no axon segmentation tool tailored to Bielschowsky-stained tissue sections exists, so a task-specific benchmark was not available; we therefore compared our model against nnU-Net v2, a strong self-configuring segmentation framework, trained on the identical data (Sect. 3.7).

On this benchmark our Attention U-Net was on Dice modestly above the nnU-Net across both the full eight-tile test set and the seven axon-bearing test tiles, while using far fewer parameters (7.9 M versus approximately 48 M). The clearest advantage of our model was on the white-matter tile 2_3 (Dice 0.805 versus 0.630), where axons run largely perpendicular to the sectioning plane and therefore appear as small cross-sectional profiles rather than elongated fibers. Given the small number of test tiles (*n* = 8), this comparison should be read as an honest benchmark rather than a definitive ranking, and it indicates that our compact, task-specific model performs on par with, or slightly better than, a fully self-configuring pipeline at a fraction of the model size.

Methodologically, we acknowledge that we trained the model in a small dataset, which bears the intrinsic risk that its performance depends on individual training examples. However, the LOWO analysis indicates that model performance is not strongly dependent on any single training WSI, as shown by the narrow Dice range across folds (0.687–0.725, SD 0.010). This suggests that the small training dataset provides complementary coverage of relevant tissue variation, and that robust segmentation can be achieved from a limited number of annotated tiles when extensive patch extraction and data augmentation are used.

## Summary

In this study, we present a deep learning-based tool for automatic axon segmentation in Bielschowsky-stained tissue sections, achieving a pixel-wise F1/Dice of 0.717 ± 0.162 on a held-out test set spanning four brains, multiple tissue classes, and both normal and demyelinated tissue — performance that meets or exceeds human inter-rater agreement. The attention U-Net architecture, trained with Focal Tversky Loss to address class imbalance, produces tile-level and whole-slide segmentation maps from which spatially resolved axon density, dominant fiber orientation, and local fiber coherency metrics can be derived. Leave-one-WSI-out analysis confirmed robustness to individual training examples (Dice range: 0.687–0.725), supporting practical utility even with limited annotated data. The pipeline is released as an open-source software package for straightforward deployment on tiled images and whole-slide scans. Future integration with multimodal registration frameworks would enable direct spatial alignment between the derived WSI orientation maps and ex-vivo or in-vivo MRI datasets, establishing a quantitative histological ground truth for microstructure imaging models and opening new avenues for bridging mesoscopic and macroscopic analyses of axonal pathology.

## Supplementary Information

Below is the link to the electronic supplementary material.

Supplementary Material 1 (DOCX 97.0 MB)

## Data Availability

Source code (version 1.0.1) is archived on Zenodo and available at https://doi.org/10.5281/zenodo.19455087. The trained model is available on Hugging Face at https://huggingface.co/lukas-schoenenberger/BasNet/tree/main. Annotated training data are deposited on a private Hugging Face repository at https://huggingface.co/datasets/lukas-schoenenberger/Basnet-dataset and are available from the corresponding author upon reasonable request with the permission of the German MS Brain Bank of the Competence Network Multiple Sclerosis.
