# Supplementary material for: BasNet: Attention U-Net-Based Automated Axon Segmentation in Bielschowsky Silver-Stained Histology
Source: Neuroinformatics. 2026 Sep 8;24(3):59. doi: 10.1007/s12021-026-09815-z (PMC13553709; doi:10.1007/s12021-026-09815-z)
Supplement: Supplementary file 1 [file 12021_2026_9815_MOESM1_ESM.docx]

**Online Resource 1**

**BasNet**: Attention U-Net-Based Automated Axon Segmentation in Bielschowsky Silver-Stained Histology

Journal: Neuroinformatics

Authors: Lukas Schönenberger^1,2^, Laurin Egli^1,2^, Dimitrios Gkotsoulias^1,2,3^, Christine Stadelmann^4,*^ & Cristina Granziera^1,2,3,*^

Affiliations: ***^1^*** *Research Center for Clinical Neuroimmunology and Neuroscience Basel (RC2NB), University of Basel, CH;* ***^2^*** *Translational Imaging in Neurology Basel, Department of Biomedical Engineering, Faculty of Medicine, University of Basel, CH;* ***^3^*** *Department of Neurology, University Hospital Basel, CH;* ***^4^*** *Department of Neuropathology, University Medical Center Göttingen, Göttingen, Germany*

***^*^*** *These authors contributed equally*

**Contact:**

Corresponding author: cristina.granziera@unibas.ch

Submitting author: lu.schoenenberger@unibas.ch

**Online resource 1:** Supplementary material containing Supplementary Results 1-3, Supplementary Tables 1-3, and Supplementary Figs. 1-6, including leave-one-WSI-out training and validation curves, ablation analyses of loss weighting and attention gates, baseline comparison against nnU-Net, and application of the trained model to coarser-resolution Bielschowsky sections.

**A**

**B**

**Supplementary Fig. 1**: Validation Loss and Training Loss over all LOWO folds. Left-out WSI per LOWO run are indicated by different colors.

**Supplementary Fig. 2:** Validation set performance over all LOWO folds. Left-out WSI per LOWO run are indicated by different colors. **A** Shows Dice score, **B** IoU**, C** Recall and **D** Precision.

**Supplementary results 1: ablation of loss weighting and attention gates**

The following supplementary items support the ablation study described in Section 3.6 of the main text (Reviewer 1, comment 1).

Supplementary Table 1: Tversky loss-weighting sweep on the held-out test set (attention gates enabled, γ = 2.0, α = 1 − β). Values are the mean across the 8 test tiles. The highest-scoring setting (β = 0.6), used in the main model, is shown in bold.

| **β** | **α** | **Dice** | **IoU** | **Precision** | **Recall** |
| --- | --- | --- | --- | --- | --- |
| 0.1 | 0.9 | 0.587 | 0.450 | 0.887 | 0.472 |
| 0.2 | 0.8 | 0.645 | 0.507 | 0.824 | 0.552 |
| 0.3 | 0.7 | 0.669 | 0.531 | 0.819 | 0.589 |
| 0.4 | 0.6 | 0.705 | 0.574 | 0.769 | 0.687 |
| 0.5 | 0.5 | 0.702 | 0.569 | 0.737 | 0.696 |
| **0.6** | **0.4** | **0.715** | **0.582** | **0.721** | **0.743** |
| 0.7 | 0.3 | 0.713 | 0.575 | 0.623 | 0.869 |
| 0.8 | 0.2 | 0.705 | 0.567 | 0.630 | 0.824 |
| 0.9 | 0.1 | 0.666 | 0.523 | 0.552 | 0.882 |

Supplementary Fig. 3: Tversky weighting sweep. Test Dice, IoU, and F1 (mean ± SD across the 8 test tiles) as a function of β (with α = 1 − β, γ = 2.0). The dashed line marks the selected value β = 0.6.

Supplementary Fig. 4: Attention-gate ablation at α = 0.4, β = 0.6. Test metrics (mean ± SD across three seeds) with and without attention-gated skip connections.

**Supplementary results 2: baseline comparison against nnU-Net**

The following supplementary items support the baseline comparison described in Section 3.7 of the main text.

Supplementary Table 2: Comparison of the Attention U-Net (ours) and nnU-Net v2 on the held-out test set, scored with the identical stratified per-tile protocol (mean ± SD across tiles; binarization threshold 0.5). Results are shown for all eight test tiles and for the seven axon-bearing tiles after excluding the background tile 3_4, whose empty ground truth yields a degenerate Dice. Paired Wilcoxon signed-rank on per-tile Dice: p = 0.023 (8 tiles) and p = 0.047 (7 tiles).

| **Model** | **Dice** | **IoU** | **Recall** | **Precision** |
| --- | --- | --- | --- | --- |
| **All eight test tiles** | | | | |
| Attention U-Net (ours) | 0.717 ± 0.162 | 0.584 ± 0.201 | 0.754 ± 0.201 | 0.717 ± 0.131 |
| nnU-Net v2 | 0.555 ± 0.245 | 0.417 ± 0.200 | 0.655 ± 0.246 | 0.655 ± 0.259 |
| **Seven axon-bearing tiles (excluding background tile 3_4)** | | | | |
| Attention U-Net (ours) | 0.677 ± 0.130 | 0.524 ± 0.134 | 0.719 ± 0.191 | 0.676 ± 0.080 |
| nnU-Net v2 | 0.634 ± 0.135 | 0.477 ± 0.132 | 0.606 ± 0.224 | 0.749 ± 0.081 |

Supplementary Table 3: Per-tile test-set metrics for the Attention U-Net (ours) and nnU-Net v2 (patch-pooled per tile, 254 patches each, threshold 0.5). Tile IDs follow the brain_block convention of the main text (Section 2.4.1); tissue type in parentheses (WM white matter, GM grey matter, L lesion/other, BG background). Tile 3_4 is the background tile with empty ground truth.

| **Tile (type)** | **Model** | **Dice** | **IoU** | **Recall** | **Precision** |
| --- | --- | --- | --- | --- | --- |
| 1_2 (WM) | Attention U-Net (ours) | 0.699 | 0.537 | 0.691 | 0.707 |
|  | nnU-Net v2 | 0.702 | 0.541 | 0.686 | 0.719 |
| 1_3 (GM) | Attention U-Net (ours) | 0.724 | 0.567 | 0.737 | 0.711 |
|  | nnU-Net v2 | 0.719 | 0.562 | 0.745 | 0.695 |
| 2_3 (WM) | Attention U-Net (ours) | 0.805 | 0.674 | 0.857 | 0.759 |
|  | nnU-Net v2 | 0.630 | 0.460 | 0.482 | 0.910 |
| 3_4 (BG) | Attention U-Net (ours) | 1.000 | 1.000 | 1.000 | 1.000 |
|  | nnU-Net v2 | 0.000 | 0.000 | 1.000 | 0.000 |
| 3_7 (GM) | Attention U-Net (ours) | 0.608 | 0.437 | 0.802 | 0.490 |
|  | nnU-Net v2 | 0.545 | 0.375 | 0.412 | 0.806 |
| 3_14 (GM) | Attention U-Net (ours) | 0.392 | 0.244 | 0.274 | 0.691 |
|  | nnU-Net v2 | 0.348 | 0.210 | 0.225 | 0.768 |
| 4_1 (L) | Attention U-Net (ours) | 0.773 | 0.630 | 0.865 | 0.698 |
|  | nnU-Net v2 | 0.759 | 0.612 | 0.917 | 0.648 |
| 4_2 (WM) | Attention U-Net (ours) | 0.736 | 0.583 | 0.807 | 0.677 |
|  | nnU-Net v2 | 0.733 | 0.579 | 0.776 | 0.695 |

Supplementary Fig. 5: Qualitative whole-tile comparison on the eight held-out test tiles (rows; labelled by brain_block and tissue type: WM white matter, GM grey matter, L lesion/other, BG background). Columns: original Bielschowsky RGB tile, ground-truth axon mask, Attention U-Net (BasNet) prediction, nnU-Net v2 prediction, and an error overlay. In the overlay, ground truth is green; single-model errors are colored (Attention U-Net false positives orange, false negatives red; nnU-Net false positives sky blue, false negatives dark blue), and pixels where both models err are grey (both false positive light grey, both false negative dark grey). Predictions are whole-tile inferences (nnU-Net via sliding-window inference). Both models recover the major axonal architecture and differ mainly on fine, low-contrast processes; on the background tile (3_4) both correctly predict no axons.

**Supplementary results 3: application at a coarser resolution**

The following item supports the discussion of the model’s scope of applicability.

**Original Tiles**

**0.35𝜇m (20x)**

**0.70𝜇m (10x)**

**Predicted Mask**

**Overlay**

Supplementary Fig. 6: The trained model applied, without any retraining, to two tiles of two different Bielschowsky sections digitized at a coarser resolution (first row 0.35µm per pixel at 20x magnification; second row 0.70 µm per pixel at 10x magnification) than the training data (0.273 µm per pixel at 20x magnification). Left: RGB input tiles; Middle: BasNet-predicted axon segmentation; Right: Predicted mask overlayed in red on top of the RGB input image. The segmentation remains visually convincing at this resolution; no quantitative evaluation was performed here, as no annotations were available at this resolution.
